# Clinical manifestations of diffuse large B-cell lymphoma that exhibits initial symptoms in the maxilla and mandible: a single-center retrospective study

**DOI:** 10.1186/s12903-022-02056-x

**Published:** 2022-01-26

**Authors:** Yasuyuki Michi, Hiroyuki Harada, Yu Oikawa, Kohei Okuyama, Takuma Kugimoto, Takeshi Kuroshima, Hideaki Hirai, Yumi Mochizuki, Hiroaki Shimamoto, Hirofumi Tomioka, Hirokazu Kachi, Jun-ichiro Sakamoto, Kou Kayamori, Tetsuya Yoda

**Affiliations:** 1 Department of Oral and Maxillofacial Surgery, Graduate School of Medical and Dental Sciences, Tokyo Medical and Dental University, 1-5-45, Yushima, Bunkyo-ku, Tokyo, 113-8549 Japan; 2 Department of Maxillofacial Surgery, Graduate School of Medical and Dental Sciences, Tokyo Medical and Dental University, 1-5-45, Yushima, Bunkyo-ku, Tokyo, 113-8549 Japan; 3 Department of Oral and Maxillofacial Radiology, Graduate School of Medical and Dental Sciences, Tokyo Medical and Dental University, 1-5-45, Yushima, Bunkyo-ku, Tokyo, 113-8549 Japan; 4 Department of Oral Pathology, Graduate School of Medical and Dental Sciences, Tokyo Medical and Dental University, 1-5-45, Yushima, Bunkyo-ku, Tokyo, 113-8549 Japan

**Keywords:** Diffuse large B-cell lymphoma, Mandibular bone, Maxillary bone, Imaging finding, Clinical feature

## Abstract

**Background:**

Diffuse large B-cell lymphoma (DLBCL) is the most common type of lymphatic tumor; however, extranodal DLBCLs that exhibit initial symptoms in the maxilla and mandible are rare. Moreover, DLBCL is clinically classified as a moderate to highly malignant lymphatic tumor that can progress rapidly; therefore, early diagnosis is crucial. However, diagnosis is difficult as the disease causes a diverse range of clinical symptoms with no characteristic imaging findings. We conducted a clinical investigation to clarify the clinical characteristics of DLBCL that exhibits initial manifestation in the maxilla and mandible.

**Methods:**

Of the 2748 patients with malignant tumors of the oral and maxillofacial region examined at our hospital during a period of 11 years between January 2006 and December 2016, 27 primary cases diagnosed with DLBCL based on the chief complaint of symptoms in the gingiva and bone of the maxilla and mandible were enrolled in this study. Evaluations were based on sex, age, whether treatment was provided by a previous physician, symptoms, duration of disease until treatment was sought, clinical diagnosis, laboratory findings, and imaging results.

**Results:**

There were 15 cases that involved the maxilla and 12 that involved the mandible. The median duration of disease until treatment was sought was 60 d (3–450 d). All cases exhibited a tumor or a mass, and hypoesthesia of the chin was confirmed in eight cases wherein the mandible was involved. The clinical stages were stage I in eight cases, stage II in ten cases, and stage IV in nine cases. Serum lactate dehydrogenase (LDH) levels were elevated in 13 of 22 patients. The overall survival rate was 63%.

**Conclusions:**

Symptoms associated with nontender swelling and numbness of the lip or chin in the absence of other findings such as dental infections should raise suspicions about DLBCL. Patients should be provided appropriate imaging and accurate biopsy assessments to improve prognosis.

## Background

Diffuse large B-cell lymphoma (DLBCL) is the most common type of lymphoma, and accounts for 30–40% of such lesions [1]. Nodal lymphomas arise within a lymph node, whereas extranodal lymphomas develop in nonlymph node tissues. Extranodal DLBCLs in the maxilla and mandible are rare, and differential diagnosis is difficult as they often exhibit clinical findings similar to that found in tumors and/or inflammation at other sites. Some reports have described the clinical characteristics of extranodal DLBCLs of the maxilla and the mandible [2, 3]. Patients with DLBCL of the oral cavity and oropharynx have low survival rates, and very few published reports describe their clinical characteristics [4–6]. In addition, the clinical manifestations of DLBCL that present in the maxilla and mandible may have features unique to their anatomical location but reports that clarify these details are lacking. Therefore, this study aims to describe symptomatic and imaging features of DLBCL in the maxilla and mandible, compare the differences in clinical presentation between the maxilla and the mandible, and report on the survival rates of these patients.

## Methods

Of the 2748 cases of malignant tumors of the mouth and jaws examined at our hospital during the 11 years from January 2006 through December 2016, 27 primary cases definitively diagnosed with DLBCL based on the chief complaint of symptoms in the gingiva and bone of the maxilla and mandible were enrolled in this study. There were 19 male and 8 female patients. The median age at the initial examination was 72 years (37–95 years). The site of onset was the maxilla in 12 cases, maxillary gingiva in 3, mandible in 10, and mandibular gingiva in 2 (Table 1).

**Table 1:** Demographic and clinical features of 27 patients

No	Anatomic location	Prior treatment	Duration of illness (days)	Swelling	Paralysis	Ulceration	Pain	Tooth mobility	PS	First clinical diagnosis
1	Maxillary gingiva	−	14	+	−	−	−	−	1	pericoronitis
2	Maxilla	−	90	+	−	−	−	+	3	gingival carcinoma
3	Maxilla	RCT	30	+	−	−	−	+	0	maxillary sinus carcinoma
4	Maxilla	RCT	60	+	−	−	+	+	0	ML
5	Maxilla	−	150	+	−	−	−	+	0	nonepithelial malignant tumor
6	Maxilla	Incision	14	+	−	−	−	+	0	inflammation
7	Maxilla	−	21	+	−	+	+	−	0	maxillary sinus carcinoma
8	Maxilla	Tooth extraction	60	+	−	−	−	−	0	nonepithelial malignant tumor
9	Maxillary gingiva	Incision	60	+	−	+	−	+	1	gingival carcinoma
10	Maxilla	−	42	+	−	+	−	edentulous jaw	3	nonepithelial malignant tumor
11	Maxilla	Tooth extraction	14	+	−	+	−	−	1	gingival carcinoma
12	Maxilla	−	14	+	−	+	+	edentulous jaw	0	ML
13	Maxillary gingiva	Injury treatment	120	+	−	−	−	edentulous jaw	2	gingival carcinoma
14	Maxilla	−	60	+	+	+	−	edentulous jaw	3	salivary gland carcinoma
15	Maxilla	Incision	60	+	+	−	−	+	0	nonepithelial malignant tumor
16	Mandibular	Operation (another diagnosis)	450	+	+	+	−	−	0	malignant tumor
17	Mandibular	Operation (another diagnosis)	360	+	+	−	−	+	0	osteomyelitis
18	Mandibular	Tooth extraction	330	+	−	−	+	−	0	mandibular tumor
19	Mandibular	−	3	+	+	−	+	−	0	carcinoma (PIOSCC)
20	Mandibular	Tooth extraction	210	+	−	−	−	−	1	inflammatory granulation tissue
21	Mandibular	RCT	90	+	+	−	+	+	1	osteomyelitis
22	Mandibular gingiva	Tooth extraction	6	+	−	−	−	+	1	malignant tumor
23	Mandibular	Tooth extraction	14	+	+	−	+	+	0	ML
24	Mandibular	−	7	+	+	−	+	−	0	ML
25	Mandibular	−	150	−	+	−	+	−	0	ML
26	Mandibular gingiva	Incision	150	+	−	+	+	−	1	mandibular tumor
27	Mandibular	−	90	+	+	−	+	−	0	ML

M: Male, F: Female, RCT: root canal treatment, PS: ECOG performance status, ML: malignant lymphoma

We investigated the parameters, including symptoms, clinical diagnosis, clinical stage, treatment method, and prognosis based on the medical records of these patients. The clinical manifestations of cases that occurred in the maxilla were compared with those in the mandible, and any differences were noted.

χ^2^ tests or Fisher’s exact probability tests were used for statistical analysis. The values were expressed as mean ± standard deviation. The Kaplan–Meier limit method was employed to determine overall survival (OS). Follow-up intervals were calculated in months from the date of the first visit to our hospital to the date of the last follow-up or death. Statistical significance was determined using log-rank (Mantel–Cox) tests. P-values < 0.05 were considered statistically significant. The analyses were performed using SPSS Statistics version 25 (IBM, Chicago, IL, USA).

This study was conducted in accordance with the Declaration of Helsinki and was approved by the ethics committee of Tokyo Medical and Dental University, Faculty of Dentistry (No. D2015-600-03). Notices about automatic opt-in consent for the study for data collection and method for opting-out were posted in the hospital, as approved by the Ethics Committee of the university. Participants were informed that there was an option for an opt-out of this retrospective research at any time by documenting the refusal of consent using the forms available.

## Results

Table 1 summarizes the clinical findings at the initial examination.

### History of treatment before the initial examination

Sixteen patients (59.3%) had previously undergone diagnosis and treatment of the site at a previous dental clinic or another Department of Oral Surgery prior to the initial examination at our department. Tooth extraction had been performed for six cases, resections for four cases, root canal treatment for three cases, and surgical treatment based on another diagnosis for two cases.

### Disease period until seeking treatment

The median disease period from the time of symptom onset to the time treatment was sought was 60 d (3–450 d). There was a significant difference between the median duration of disease until treatment was sought for maxilla (60 d) and mandible cases (120 d).

### Symptoms

Tumors were detected in almost all patients (26 cases; 96.3%). Tooth instability was noted in 11 cases (47.8%; excluding four edentulous cases), and desensitization of the chin or buccal region was present in 10 cases (2/15 maxilla cases; 13.3%, 8/12 mandible cases; 66.7%). Pain was reported by 10 patients (37.0%) and ulceration was observed in 9 cases (33.3%). The Eastern Cooperative Oncology Group (ECOG) Performance Status was ≥ 2 in five cases (18.5%).

### Clinical diagnosis at the initial examination

Based on these clinical symptoms and findings, the diagnosis made at the initial examination was suspected malignant lymphoma (ML) in 6 cases (22.2%), suspected malignant tumor in 14 cases (51.9%), suspected benign tumor in 2 cases (7.4%), and suspected inflammation in 5 cases (18.5%).

### Differences in symptoms between the maxilla and mandible

There were no statistically significant differences between the mandible and maxilla in terms of presence or absence of treatment received prior to the first visit (*P* = 0.43). Paresthesia of the buccal or lower lip region was significantly more common in mandible cases (*P* = 0.016). There were no significant differences in terms of ulcer formation between the maxilla and mandible (*P* = 0.10). Pain was more common in mandible cases, which was statistically significant (*P* = 0.015).

Table 2 summarizes the imaging findings, hematological findings, staging, and clinical course.

**Table 2:** Imaging findings and clinical course of 27 patients

	CT	MRI	PET-CT			LDH	EBV	Stage	NCCN-IPI risk group	Treatment		
No	Permeative pattern radiolucent finding	ADC (× 10^−3^ mm^2^/s)	SUV_max_	MTV	TLG					Chemotherapy	RT	
1	−	−	20	73.8	319.6	−	−	2B	L–I	R-CHOP*3	30 Gy	N.E.D
2	+	−	39.3	956.3	6378.5	376	−	4A	H	BSC		D.O.C
3	+	−	31.7	1114.2	7621.1	208	−	2EA	L–I	R-CHOP		N.E.D
4	+	0.681	6.8	24	98.4	108	−	4A	H–I	R-CHOP		N.E.D
5	+	−	−	−	−	204	−	1EA	L–I	R-CHOP		N.E.D
6	+	−	14.6	1075.6	5378	428	−	2EA	H–I	R-THP-COP		D.O.D
7	+	−	−	−	−	258	−	1EA	H–I	R-CHOP		N.E.D
8	+	−	33.2	40.5	421.2	174	−	2EA	L–I	R-CHOP		N.E.D
9	−	−	15.6	45.5	200.2	222	−	2EA	H–I	R-THP-COP		N.E.D
10	+	0.45	16.7	1022.1	5008.3	511	−	4B	H	BSC		D.O.C
11	+	0.58	32.4	121.9	889.9	187	−	2EA	L–I	R-CHOP		N.E.D
12	+	0.5	47.6	750.4	4052.2	293	−	1EA	H–I	R-THP-COP		N.E.D
13	−	0.952	13.9	51.6	180.6	142	+	2EA	H–I	R-COEP		N.E.D
14	+	−	38.5	1343.2	9402.4	368	−	2EA	H	BSC		D.O.C
15	+	0.486–0.566	68.6	269.5	5470.9	557	−	4A	H–I	R-CHOP		N.E.D
16	−	−	29.1	564.1	5979.5	268	−	1A	L–I	R-CHOP	40 Gy	N.E.D
17	+	−	6.7	-	-	×	−	1EA	L–I	CHOP	30 Gy	N.E.D
18	−	−	5.8	2.9	9.3	287	−	2A	H–I	R-CHOP		D.O.D
19	+	−	20.8	60.5	423.5	310	−	4A	H–I	R-CHOP		D.O.D
20	−	−	17	24.3	145.8	222	−	2EA	H–I	R-CHOP		N.E.D
21	−	−	16.2	37.4	235.6	278	−	4A	H	R-CHOP		N.E.D
22	−	0.735	8.7	16.2	64.8	185	+	1A	H–I	BSC		D.O.C
23	−	−	−	−	−	253	−	4EA	H–I	R-CHOP		D.O.D
24	+	0.581	15.1	1199.6	4318.56	216	−	4A	L–I	R-CHOP		N.E.D
25	+	0.648	7.2	76.6	268.1	172	+	1A	L–I	R-CHOP	30 Gy	N.E.D
26	−	−	−	−	−	155	−	1A	H–I	R-CHOP		D.O.C
27	+	0.73	7.2	40.8	146.88	247	−	4A	H–I	R-CHOP		D.O.D

N.E.D: no evidence of disease, D.O.C: died from other cause, D.O.D: died of disease

### Imaging findings

Characteristic imaging findings that indicate ML in the maxilla or the mandible include permeable bone resorption on computed tomography (CT) images, low apparent diffusion coefficient (ADC) for the mass on magnetic resonance imaging (MRI) [7], and strong fluorodeoxyglucose (FDG) uptake on positron emission tomography–computed tomography (PET–CT) [8]. In this study permeable bone resorption on CT was noted in 12/15 patients (80%) who underwent imaging of the maxilla, and 5/12 patients (41.7%) who underwent imaging of the mandible. Permeable bone changes were observed when the base of the tumor was in the mandibular body or ramus.

In contrast to those with other tumors, in some cases with marked progression into the bone of the maxilla, or the mandible, or progression into the maxillary sinus with permeable bone resorption, a return to almost normal anatomical structure was confirmed after treatment. The anatomical structure did not recover completely in cases that had progressed into the alveolar bone due to teeth movement. Resorption of the alveolar bone was common on the buccal side of the mandible, in particular, in cases with inflammation. Many patients with lesions in the alveolar bone exhibited compression-type bone resorption.

The median ADC was 0.62 × 10 − 3 mm^2^/s for the 10 cases wherein confirmatory assessment was performed using MRI. FDG uptake on PET–CT was observed in all patients who underwent such testing, and the median standardized uptake value (SUV_max_) was 16.7. The FDG uptake was stronger in patients whose maxillae were involved compared with those whose mandibles were involved. The mean maximum SUV_max_ was 29.1 for the cases wherein maxillae were involved, and 13.4 for those whose mandibles were involved. The SUV_max_, metabolic tumor volume (MTV), and total lesion glycolysis (TLG) increased with larger target lesions, and the values were higher for patients whose maxillae were involved compared with those whose mandibles were involved.

### Hematological findings

Serum lactate dehydrogenase (LDH) and soluble interleukin-2 receptor (sIL-2R) are the biomarkers for ML. Serum LDH was higher than the normal upper limit in 13 patients. The measurement of sIL-2R was performed only for three patients, out of which one patient exhibited an abnormally high value for sIL-2R.

### Clinical stage, National Comprehensive Cancer Network–International Prognostic Index (NCCN-IPI), treatment method, and prognosis

Clinical stage, as per the Ann–Arbor staging system, was stage I for 8 patients, stage II for 10 patients, and stage IV for 9 patients. B symptoms (fever, night sweats, and weight loss) were observed in one stage II case and one stage IV case.

**Fig. 1:**
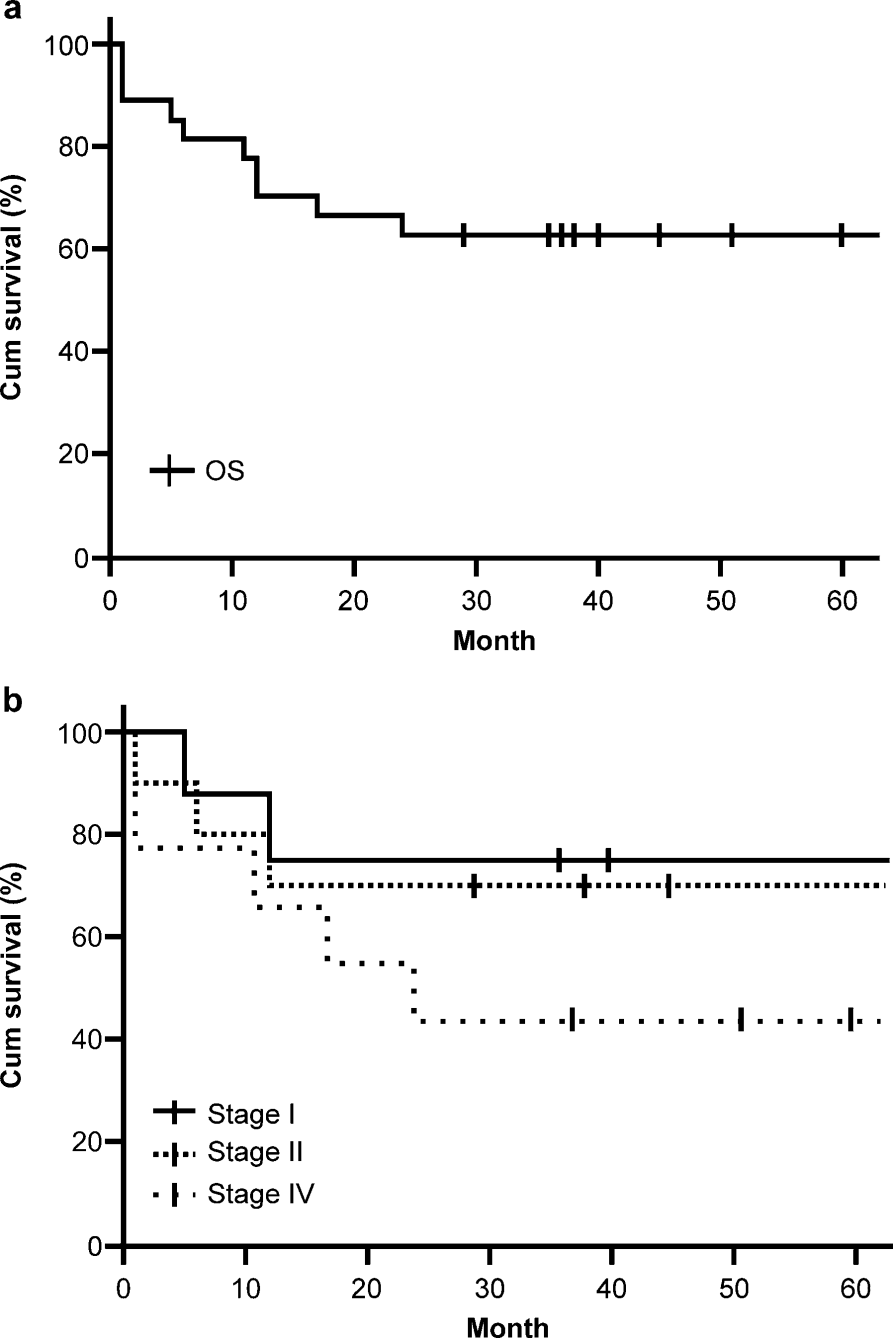
Kaplan–Meier survival analysis in patients with DLBCL. **a** Representation of total 5-year overall survival (OS) in patients with DLBCL. **b** OS in patients with DLBCL according to Ann–Arbor classification

NCCN-IPI results based on age (≥ 60 years), serum LDH, performance status (PS) (ECOG PS2–4), clinical staging (III, IV), and at least two extranodal lesions was low-intermediate for 9 patients, high-intermediate for 14 patients, and high for 4 patients. The treatment method was 6–8 courses of chemotherapy based on R-CHOP for 19 patients, 3–4 courses of R-CHOP and radiotherapy at 30–40 Gy applied to the head and neck region for 4 patients, and best supportive care (BSC) for 4 patients. Reasons for BSC included difficulty in the treatment procedure, patient refusal to undergo treatment, or dementia. The 5-year survival rate was 63% (Fig. 1a) for the overall study population (n = 27), 75% for stage I patients, 70% for stage II patients, and 44% for stage IV patients (Fig. 1b). All the patients in the group classified as high as per the NCCN-IPI were elderly; among them only one patient could undergo chemotherapy, and all patients had a poor prognosis. All the patients in the low-intermediate group demonstrated disease-free survival. The sample size was relatively small; therefore, no significant differences were noted among the groups.

### Case presentation

**Fig. 2:**
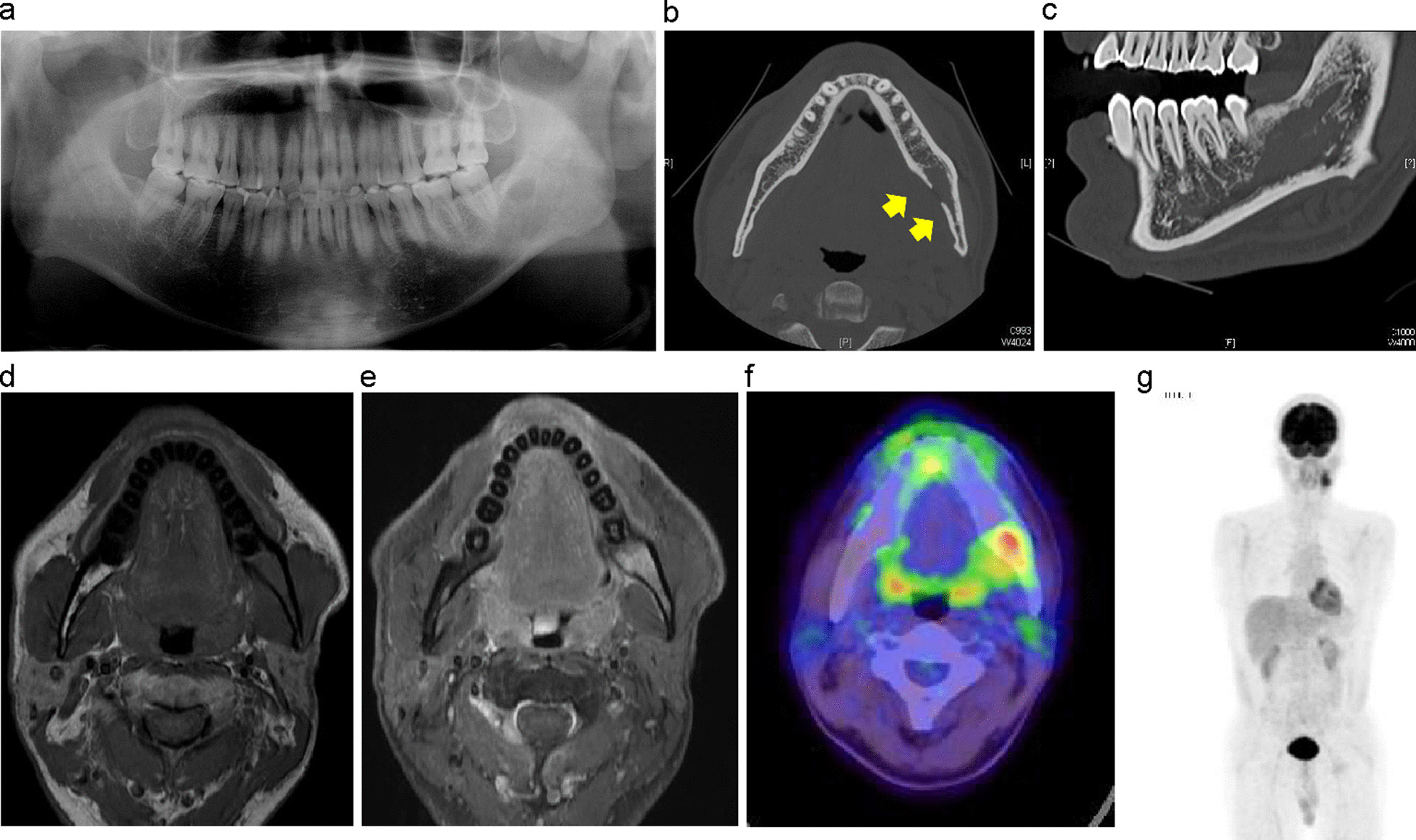
Case 1. A 45-year-old man with DLBCL, stage IA. **a** Panoramic radiograph image taken at the first visit showing a radiopaque area in the mandibular left third molar area. **b** Axial CT scan using a bone algorithm at the first visit showing penetrating resorption of the cortical bone in the lingual aspect of the left mandible (yellow arrows). **c** Sagittal CT scan image showing marginal resorption of the mandibular canal wall. **d** Contrast-enhanced MRI of the bone marrow of the mandible showed low signal intensity on T1-weighted image. **e** Homogeneous enhancement on post-contrast fat-suppressed T1-weighted image. **f** Fluorodeoxyglucose PET/CT scan. **g** Maximum intensity projection image

*Case* 1 (Patient no. 25): A 45-year-old man presented to the Department of Oral and Maxillofacial Surgery at Tokyo Medical and Dental University with a 5-month history of pain in the mandibular left molar area, paralysis of the left mentum region, and swelling of the left side of the mandible. A panoramic radiograph showed a radiopaque area in the mandibular left third molar region measuring 3 cm in length (Fig. 2a). A CT scan of the left side of the mandible showed penetrating resorption of the lingual cortical bone and marginal resorption of the mandibular canal wall without periosteal reaction or osteosclerosis (Fig. 2b, c). Contrast-enhanced MRI showed an osteolytic lesion in the left side of the mandible with low signal on T1-weighted images and relatively homogeneous enhancement on post-contrast fat-suppressed T1-weighted images (Fig. 2d, e). PET–CT showed a 39 × 25 mm lesion with SUV_max_ of 7.2 in the left posterior mandible; no other FDG accumulation was identified in the cervical lymph nodes or elsewhere in the body (Fig. 2f, g). Blood test results showed LDH and sIL-2R to be within normal limits (172 U/L and 458 U/mL, respectively), and no other abnormalities were observed. After three courses of R-CHOP therapy and 30 Gy of external radiation, the lesion disappeared.

**Fig. 3:**
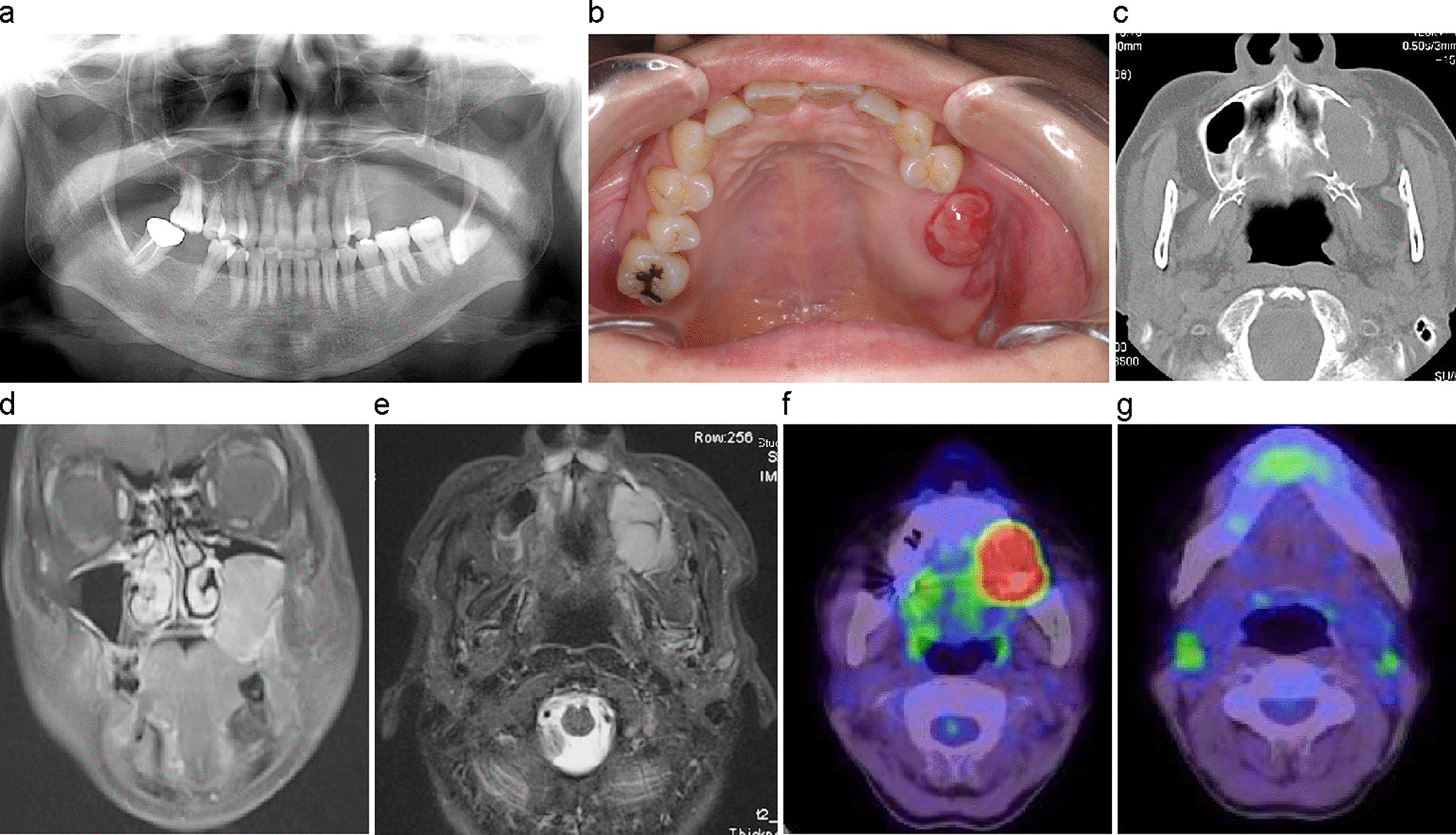
Case 2. A 59-year-old female with DLBCL, stage II_E_A. **a** Panoramic radiograph image taken at the initial visit showing bone loss at the base of the maxillary left sinus. **b** Intraoral view of the maxilla, initial visit. **c** Contrast-enhanced CT showing a neoplastic lesion located in the maxillary bone in the maxillary left sinus region with permeable resorption of the surrounding bone. **d** Contrast-enhanced MRI showing a mass with low signal on T1-weighted images. **e** Contrast-enhanced MRI showing high-signal mass on T2-weighted images. **f** Fluorodeoxyglucose PET/CT showing a lesion with SUV_max_ of 33.2. **g** Fluorodeoxyglucose accumulation of up to 3.7 in the right cervical lymph node

*Case* 2 (Patient no. 8): A 59-year-old female was referred to the Department of Oral and Maxillofacial Surgery at Tokyo Medical and Dental University with the main complaint of swelling of the maxilla of 2 months' duration. One month earlier she had undergone extraction of the maxillary left first and second molars. The extraoral exam revealed no abnormalities, or paralysis of the maxillary, or facial nerves. Panoramic radiographs showed bone loss at the base of the maxillary left sinus in the region corresponding to tooth nos. 5–7, and irregular bone resorption at the margins (Fig. 3a). The maxillary left molar area was distended, and a granulomatous mass that bled easily was found in the center of the mass (Fig. 3b). Contrast-enhanced CT showed a neoplastic lesion occupying the maxillary bone and extending to the maxillary left sinus region, with permeable resorption in the surrounding bone (Fig. 3c). Contrast-enhanced MRI showed a mass with low signal on T1-weighted images and high signal on T2-weighted images (Fig. 3d, e). PET–CT showed a lesion with SUV_max_ of 33.2 in the left maxilla extending to the maxillary sinus as well as FDG accumulation of up to 3.7 in the cervical lymph nodes (Fig. 3f, g). A biopsy of the granulosa-like mass was performed, and a histopathological diagnosis of DLBCL was made. Following six courses of R-CHOP, the patient was judged to be in complete remission, with no subsequent recurrence.

## Discussion

ML is the third most common malignant lesion of the oral cavity and the maxillofacial region after squamous cell carcinoma and salivary gland cancer [8, 9]. Malignant lymphomas can broadly be classified based on histopathological findings as either Hodgkin’s lymphoma or non-Hodgkin’s lymphoma [10]. A majority of the lymphomas that develop in the oral cavity region are non-Hodgkin’s lymphomas.

DLBCL, not otherwise specified as defined in the 2017 World Health Organization (WHO) classification, accounts for > 30% of all non-Hodgkin lymphomas in Japan, making it the most prevalent form of NHL [11]. Approximately 40% of DLBCLs involves extranodal lesions [12]. Oral cavity DLBCLs mainly arise in the gingival and palate mucosa, while a few studies have reported that such lesions arise in the jawbone [13]. Our data indicated that most of these cases arose in the jawbone, whereas few developed in the gingiva.

Lesions were more common in males compared with those in females (2.4:1), with a higher proportion of male cases being reported than in previous trials [1, 14, 15]. The mean age in our study (69 years) was equivalent to that of earlier studies [1, 14, 15].

As DLBCLs that arise in the jawbone also often involve dental infections, many patients undergo treatments, such as root canal therapy and periodontal treatment [13]. We found that 59.3% of our patients had undergone some type of dental treatment before the initial examination performed at our department.

Clinical symptoms are diverse, including painless tumors, tooth instability, desensitization of the buccal or chin region, and ulceration. Most patients are asymptomatic in the initial stages, and various symptoms begin to appear as the lesion increases in size. This could be the reason for the high proportion of clinical misdiagnosis and delayed diagnosis [16].

Comparing the cases that occurred in the maxilla and mandible, we found that most of the former produced painless swelling, whereas most of the latter produced numbness of the lip, or chin area. Such symptoms, for which no dental infections, or other causes can be found, should raise suspicion about malignancy. Halt any invasive dental procedures that may be planned, and prompt immediate referral to an oral and maxillofacial surgeon for appropriate imaging and accurate biopsy testing.

With respect to the imaging findings, bone destruction was not clearly observable on panoramic radiography images; however, careful observation revealed diffuse bone destruction, a finding that is not common in ordinary dental infections or other neoplastic diseases. Additionally, the maxillary sinus border in the maxilla appeared to have vanished, and the cortical bone in the mandible appeared unclear, with increased radiolucency. Previous reports have shown that on CT images, relatively little cortical bone destruction will be observable, with masses that exhibit a permeative pattern of bone destruction with no clear periosteal reaction [17]. In our study, 80% of the cases with maxilla involvement exhibited permeable bone resorption on CT images, a much higher percentage than was found in cases with mandible involvement. We believe that this is reflective of the fact that the tumor diameters in the cases with maxilla involvement were larger than that for those with mandible involvement. Hypointense signals on T1-weighted MRI, and moderate enhancing effects on fat-suppressed contrast-enhanced T1-weighted MRI are common observations. In jawbone DLBCL, ADC is low on diffusion-weighted images, and in contrast to many other squamous cell carcinomas in the oral cavity, strong diffusion is observed [18]. On FDG-PET, FDG uptake localized to the tumor region was observed. Similar to what is observed in other tumors, SUV_max_ was unrelated to malignancy or prognosis, and was dependent on tumor size. SUV_max_ was smaller for patients with maxilla involvement and a large tumor diameter (median: 42 mm) than for those with mandible involvement and a small tumor diameter (median: 33 mm).

The serum LDH activity and sIL-2R levels are measured as biomarkers for lymphoma patients [19]. However, these are rarely measured in patients who are not initially diagnosed with ML in the clinical setting. The LDH levels were elevated in approximately 50% of our patients who underwent hematological testing in the early stages. The serum LDH levels often rise nonspecifically; therefore, we believe that it should be used as an auxiliary aid for diagnosis.

Many DLBCLs of the oral cavity and the maxillofacial region are believed to be stage I or II at the onset [15]; however, approximately one-third of our patients were classified into stage IV. This ratio was higher than what has been reported in previous studies. B symptoms are generally uncommon and were noted only in 7.4% of our patients. While the OS of 63% cannot not be described as highly favorable, it is consistent with previous reports. NCCN-IPI results closely reflected the prognosis. In the future, treatment methods for patients with poor prognosis need to be developed.

## Conclusions

Symptoms associated with painless swelling and numbness of the lip or chin that cannot be attributed to dental infections, or other findings should raise suspicions about DLBCL. Such patients should not be scheduled for any invasive dental procedures, and instead should be immediately referred to an oral and maxillofacial surgeon for appropriate imaging and accurate biopsy testing. These steps should allow for early and accurate diagnosis of DLBCL that presents in the jaw, and will lead to improvements in prognosis.

## Data Availability

The data is available from the corresponding author through e-mail.

## Abbreviations

DLBCL: Diffuse large B-cell lymphoma
ML: Malignant lymphoma
ECOG: Eastern Cooperative Oncology Group
CT: Computed tomography
ADC: Apparent diffusion coefficient
MRI: Magnetic resonance imaging
FDG: Fluorodeoxyglucose
PET/CT: Positron emission tomography–computed tomography
SUV_max_: Maximum standardized uptake value
MTV: Metabolic tumor volume
TLG: Total lesion glycolysis
LDH: Lactate dehydrogenase
sIL-2R: Soluble interleukin-2 receptor
NCCN-IPI: National Comprehensive Cancer Network–International Prognostic Index
PS: Performance status
BSC: Best supportive care
WHO: World Health Organization
NHL: Non-Hodgkin lymphomas
EBV: Epstein-Barr virus
